# Prevalence and dynamics of clinically significant bacterial contaminants in herbal medicines sold in East Africa from 2000 to 2020: a systematic review and meta-analysis

**DOI:** 10.1186/s41182-020-00295-8

**Published:** 2021-01-28

**Authors:** Abdul Walusansa, Savina Asiimwe, Hussein. M. Kafeero, Iramiot. J. Stanley, Jamilu. E. Ssenku, Jesca. L. Nakavuma, Esezah. K. Kakudidi

**Affiliations:** 1 Department of Plant Sciences, Microbiology and Biotechnology, School of Biosciences, Makerere University, Kampala, Uganda; 2 Department of Medical Microbiology and Immunology, Faculty of Health Sciences, Busitema University, Mbale, Uganda; 3 Department of Microbiology and Immunology, Faculty of Health Sciences, Habib Medical School, Islamic University in Uganda, Kampala, Uganda; 4 Department of Biomolecular and Biolaboratory Sciences, College of Veterinary Medicine, Animal Resources and Biosecurity, Makerere University, Kampala, Uganda

**Keywords:** Bacterial contamination, Herbal medicine, East Africa, Systematic review

## Abstract

**Background:**

Infectious diseases remain a leading cause of mortality and morbidity around the world, and those caused by bacteria are common in the East African region. In this region, trade and consumption of herbal medicine has been expanding in the recent decades. Herbal medicines may be contaminated with pathogenic bacteria; however, there is limited information due to fragmented studies in East Africa. In this meta-analysis, we critically analyzed original research related to the incidence of pathogenic bacterial contaminants of HM in the East African region since 2000. The aim was to create a comprehensive understanding of the extent and dynamics of bacterial contamination in HM, to guide future research and concerted public health protection in the region.

**Methodology:**

The study was conducted according to the standards of the Preferred Reporting Items for Systematic Reviews and Meta-analyses. We searched and evaluated published articles from eleven electronic databases (Google Scholar, PubMed, HerbMed, MEDLINE, Science Direct, Scifinder Scholar, Cochrane Library, International Pharmaceutical Abstracts, EMBASE, Biological Abstracts and Commonwealth Agricultural Bureau Abstracts). Prevalences of different bacterial species, Cochran’s *Q* test, and the *I*^2^ statistic for heterogeneity were evaluated using a software called MedCalcs. Random and fixed effects models were used to determine the pooled prevalence of clinically significant bacteria from studies which were included in this meta-analysis. The potential sources of heterogeneity were examined through sensitivity analysis, sub-group analysis, and meta-regression at 95% level of significance.

**Results:**

Fourteen studies met our inclusion criteria. Overall, the studies were highly heterogeneous (*I*^2^ = 98.48%) and there was no evidence of publication bias. *Escherichia coli* was the most prevalent contaminant. *Salmonella* spp. and *Shigella* spp. were the most frequently reported primary pathogens with pooled prevalence of 10.4% and 6.3%, respectively. Our findings are in tandem with recent systematic reviews conducted in Europe and Asia, but are in discrepancy with the reviews recently conducted in southern Africa.

**Conclusion and recommendations:**

The East African herbal medicine industry poses considerable health risks to communities through dissemination of clinically significant bacteria. Presence of enteric bacterial contaminants indicates possible fecal pollution of herbal medicine region-wide. Adequate research pertaining to microbial safety of herbal medicine in the East African countries remains highly desired. The latter will enable establishment of strong, region-wide herbal safety mechanisms in order to support comprehensive public health protection in East Africa.

## Introduction

Botanical therapies are ranked among the world’s most frequently used medications [1]. The global market value of herbal medicine (HM) is now estimated at USD 71.17 billion, and anticipated to increase with a compound annual growth rate of 5.5% by the year 2027 [2]. In the East African countries, the prevalence of use of herbal medicine (HM) is reported to be over 70%, and it is used in management of health complications ranging from instant medical emergencies such as snakebite envenomation, to chronic conditions like infertility, kidney diseases, cancer, diabetes, ulcers, and HIV/AIDS-related symptoms among others, [3–7]. The ongoing increase in global consumption and trade of herbal medicine has raised safety concerns in many regions including East Africa. These concerns are mainly related to microbial contamination, heavy metal, and phytochemical toxicities [8]. Pathogenic microbial contaminants in HM have created greater anxiety due to their causation of deadly infectious diseases [9–11]. In most parts of the world, bacteria have been reported among the major pathogenic microbes that contaminate herbal medicines sold especially in urban settings [11–13]. Apart from East Africa, enormous analytical research and case reports about harmful bacterial contaminants in HM have been published, and their findings have been evaluated in a number of scientific reviews [11, 14].

In Europe, systematic reviews on HM safety by Ulbricht et al. and Basch et al. reported *Listeria monocytogenes*, *Clostridium perfringens*, *Salmonella* spp., and *Escherichia coli* among the frequently reported contaminants [11, 15]. Bacterial infections linked to the consumption of contaminated HM were also diagnosed in the population in Europe [11]. In Southern Africa, numerous bacteria, such as *Salmonella* spp., *Klebsiella pneumoniae*, *Pseudomonas spp*., *Acinetobacter baumannii*, and methicillin-resistant and vancomycin-resistant *staphylococcus aureus*, which have potential to impair human health have been reported in commercial HM [16]. In addition, bacterial toxins such as *Bacillus cereus* diarrheal toxin have also been reported [16]. In Eastern Africa, minimal, fragmented research on microbial safety of HM has been carried out within individual countries. This impedes the design of concerted herbal safety guidelines and public health policies. Our systematic review thus evaluated original research articles concerning the loads and diversity of potentially pathogenic bacterial contaminants in HM that were published between the year 2000 and 2020 in the six countries which constitute the East African community (EAC). The aim was to create a comprehensive understanding of the extent of bacterial contamination in commercial herbal medicines in East Africa, in order to support the design of concerted public health interventions and to guide subsequent herbal safety research across the East African region.

## Materials and methods

### Search strategy

We searched eleven electronic databases for published articles relating to microbial contamination of herbal medicine in the six countries which constitute the East African community (EAC) [17]. The databases that were searched and the search terms used are shown in (Table 1) below. In addition, a manual search was conducted by checking the references cited by already identified eligible publications.

**Table 1:** Databases searched and the search terms used to identify publications on bacterial contamination of herbal medicines in the EAC member countries since 2000

Database searched	Search terms
Google scholar	Herbal medicine, Indigenous traditional medicine, Microbial contamination, Bacteria, Herbal medicine safety, Herbal medicine risks, East Africa, Uganda, Tanzania, Kenya, Rwanda, Burundi, Southern Sudan.
PubMed	
HerbMed	
MEDLINE	
Science Direct	
Scifinder scholar	
Cochrane Library	
International Pharmaceutical Abstracts	
EMBASE	
Commonwealth Agricultural Bureau Abstracts	
Biological Abstracts	

All the search terms in (Table 1) above were used in each of the databases. The terms were initially used singly and then combined using linking words like, “with”, “and”, “or”, “plus”. Hand searches were also conducted to find relevant articles from journals that are not indexed in common databases. This meta-analysis was delimited to articles that were published in the past 20 years; hence, the search was limited to articles published between January 2000 and July 2020. The total output from all the databases was 23,300 results.

### Selection criteria

Initially, all published literature related to microbial contamination of herbal medicines in E. Africa was collected regardless of the research design and quality, and the attributes of the herbal samples such as diseases treated, formulation, precautions, dosing storage, and adverse effects. Final selection and inclusion of the publications was done using standardized protocols. Studies that were included met the following conditions: they must have performed enumeration and identification of bacterial contaminants in commercial HM in E. African countries, full text articles published in peer reviewed journals between 2000 and 2020, and in the English language. Exclusion was based on research conducted in African countries other than EAC, review articles, those investigating other microbial contaminants and adulterants, and those published before 2000.

### Review process

#### Data extraction from the journal articles

Four reviewers (AW, SA, HMK, and IJS) for the selection criteria are aforementioned above. The four reviewers extracted data independently from 14 articles which met the selection criteria above. Each researcher individually entered the data in spread sheets, capturing these attributes: first author, year of publication, country, mode of administration, formulation, disease(s) treated, sample size, sampling techniques, number of samples contaminated with bacteria, potentially harmful bacteria isolated, and the bacteriological methods used.

#### Quality assessment

Quality assessment for the eligible studies was independently performed by four reviewers (AW, SA, HMK, and IJS), and a quality score ranging from 0 to 10 was awarded to each study. Quality scoring was done basing on three dimensions namely sample collection, comparability, outcome, and statistical analysis, as described in guidelines of the Newcastle-Ottawa scale [18]. Studies with a score of 9–10 were described as very good, 7–8 as good study, 5–6 as satisfactory study, and less than 5 as unsatisfactory. Consistence in quality assessment of the articles was supervised by three co-authors not involved in the scoring (JES, JLN, and EKK).

#### Data analysis

The number of eligible studies and combined frequencies of clinically significant bacterial species reported in the research articles and proportions of herbal medicine samples contaminated with bacteria were evaluated and presented using bar graphs and histograms. A random effects model was used to determine the pooled prevalence of clinically significant bacteria from the studies where heterogeneity was high; however, a fixed effects model was used in cases were heterogeneity of the respective studies was low [19]. The results were presented using forest plots. Pooled prevalences were compared for association with different variables during the sub-group analysis and the *P* values at 95% CI were determined. Cochran’s *Q* test and the *I*^2^ statistic were evaluated to examine the heterogeneity of the eligible studies for our meta-analysis. Publication bias was examined by constructing funnel plots. Sources of heterogeneity of the eligible studies were assessed by conducting sensitivity analysis, sub-group analysis, and meta-regression. All the analyses were performed using statistical software called MedCalcs (https://www.medcalc.org/), and *P* < 0.05 was considered significant in all cases.

## Results

### Screening for eligible studies

**Fig. 1:**
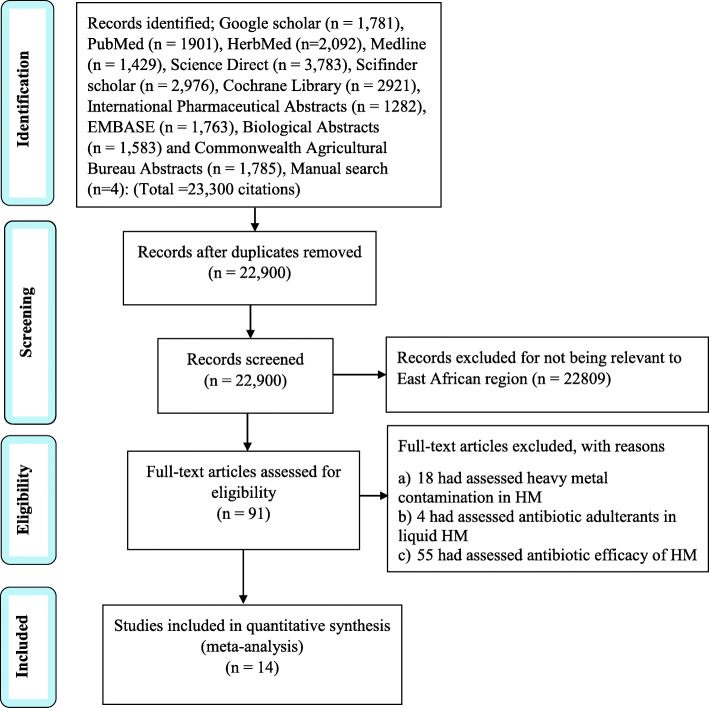
Flow chart for study eligibility screening of the research articles related to bacterial contamination of herbal medicines in East Africa, following PRISMA criterion

The PRISMA search strategy was used to screen for eligibility of the published articles concerning bacterial contamination of herbal medicines in Eastern Africa between 2000 and 2020 (Fig. 1) below. Fourteen research articles with a total sample size of 1350, of which 770 samples (57%) were positive for bacterial contamination, met our inclusion criteria.

There was no recent study that suited our inclusion criteria in Rwanda, South Sudan, and Burundi. The distribution of the eligible studies is shown in (Table 2) below.

**Table 2:** Distribution of eligible research articles on bacterial contamination of herbal medicines sold in East African countries from 2000 to 2020

Country of study	Number of studies
Uganda	4
Rwanda	0
Burundi	0
Kenya	8
Southern Sudan	0
Tanzania	2

### Characteristics of the eligible studies

Characteristics of the 14 studies included in our meta-analysis have been summarized in (Table 3). Briefly, Kenya had the highest number of eligible studies eight (57%), with a total sample size of 607; followed by Uganda with four (29%) studies, total sample size 506; and Tanzania with two (14%) studies, total sample size 159 [8, 12, 13, 20–28]. A study by Nanyanzi, conducted at Makerere University School of Public Health in Uganda, had the largest sample size of 216 [20]. The study by Musoke, at Kyambogo University in Uganda, had the smallest sample size of 20 [29]. Most studies used purposive sampling eight (57%), followed by entire sampling for five (36%), and one (7%) used simple random sampling. All the studies used convectional culture methods to isolate bacterial contaminants from the herbal medicine samples and morphological, gram-staining, biochemical, and physiological techniques to characterize the bacterial isolates. Most of the eligible studies included in this meta-analysis were published between the year 2011 and 2020; 13/14, 93%, with a total sample size of 1134; *n* = 1350, 84% [8, 12, 13, 22–30] compared to the studies published between 2000 and 2010; 1/14, 7%, with a total sample size of 216; *n* = 216, 16% [20].

**Table 3:** Characteristics of the articles concerning bacterial contamination of HM in E. Africa that were reviewed in this study

First author, year	Year	Country	ROA	Formulation	Diseases treated	Sampling technique	Sample size	BCS	Study design	Bacteria isolated
Sousa Lima et al., 2020	2020	Kenya	Oral and topical	Liquid, semi-solid, solid	Not specified	Entire	132	68	Cross-sectional	*S*. *aureus*, *Salmonella* spp., *E*. *coli*, *P*. *aeruginosa*
Ngari, F.W., et al., 2013	2013	Kenya	Oral	Solid, liquid, semi-solid	Oral diseases	Entire	22	5	Cross-sectional	*E*. *coli*, *Salmonella typhi*, *P*. *aeruginosa*
Wakome, M. J, 2015	2015	Kenya	Oral and topical	Solid, liquid	Not specified	Entire	150	142	Cross-sectional	*Klebsiella pneumonia*, *Escherichia coli*
Khadijah. M. H, 2015	2015	Kenya	Oral	Solid, liquid	HIV, TB, ulcers, cancer	Purposive	89	29	Cross-sectional	*Salmonella* spp., *E*. *coli*
Keter, K. L., et al., 2016	2016	Kenya	Oral	Solid, liquid	Not specified	purposive	100	90	Cross-sectional	K. *pneumonia*, *Shigella sonnei*, *Serratia erwinia*, *Serratia liquefaciens*, *Proteus penneri*
Kaume, L., et al., 2012	2012	Kenya	Oral	Solid	HIV	Purposive	24	16		*E*. *coli*, *S*. *aureus*
Onyambu, 2013	2013	Kenya	Not specified	Liquid, solid	Not specified	Purposive	30	23	Cross-sectional	*E*. *coli*, *K*. *pneumoniae*, *Enterobacter aerogenes*, *S*. *aureus*, *Salmonella* spp., *Shigella* spp.
Korir, R. et al., 2017	2017	Kenya	Not specified	Liquid, semi-solid, solid	Not specified	Purposive	138	117	Cross-sectional	*Klebsiella pneumoniae*, *Bacillus anthracoides*, *Proteus penner*, *S*. *aureus*, *Streptomyces* spp., *E*. *coli*, *Enterobacter aerogens*, *Serratia* spp., *Yersinia enterocolitica*, *Citrobacter* spp., *Shigella sonnei*
Walther, C., et al., 2015	2015	Tanzania	Not specified	Liquid	Not specified	Purposive	109	89	Cross-sectional	*Klebsiella* spp., *Enterobacter* spp.
Kira, J. D, 2015	2015	Tanzania	Not specified	Solids, liquids	Not specified	Random	50	9	Cross-sectional	*Escherichia coli*, *S*. *aureus*
Nanyanzi, J., 2009	2009	Uganda	Oral	Liquids	Cough	Purposive	216	24	Cross-sectional	*Escherichia coli*, *Salmonella* spp.
Musoke, W., 2019	2019	Uganda	Oral	Liquid, solid	Cough, ulcer, diabetes, malaria	Purposive	20	18	Cross-sectional	*S*. *aureus*, *E*. *coli*
Niyoshima. D., 2016	2016	Uganda	Not specified	Liquid	Not specified	Purposive	170	40	Cross-sectional	*Staphylococcus aureus*
Gonsha, R., 2012	2012	Uganda	Oral	Liquid	Not specified	Entire	100	100	Cross-sectional	*S*. *epidermidis*, *S*. *aureus*, *Enterobacter* spp., *E*. *coli*, *Citrobacter freundii*

*ROA* route of administration, *BCS* bacteriologically contaminated samples, *P*. *aeruginosa Pseudomonas aeruginosa*, *E*. *coli Escherichia coli*, *S*. *aureus Staphylococcus aureus*

### Prevalence of clinically significant bacteria in commercial herbal medicines in East African countries between 2000 and 2020

**Fig. 2:**
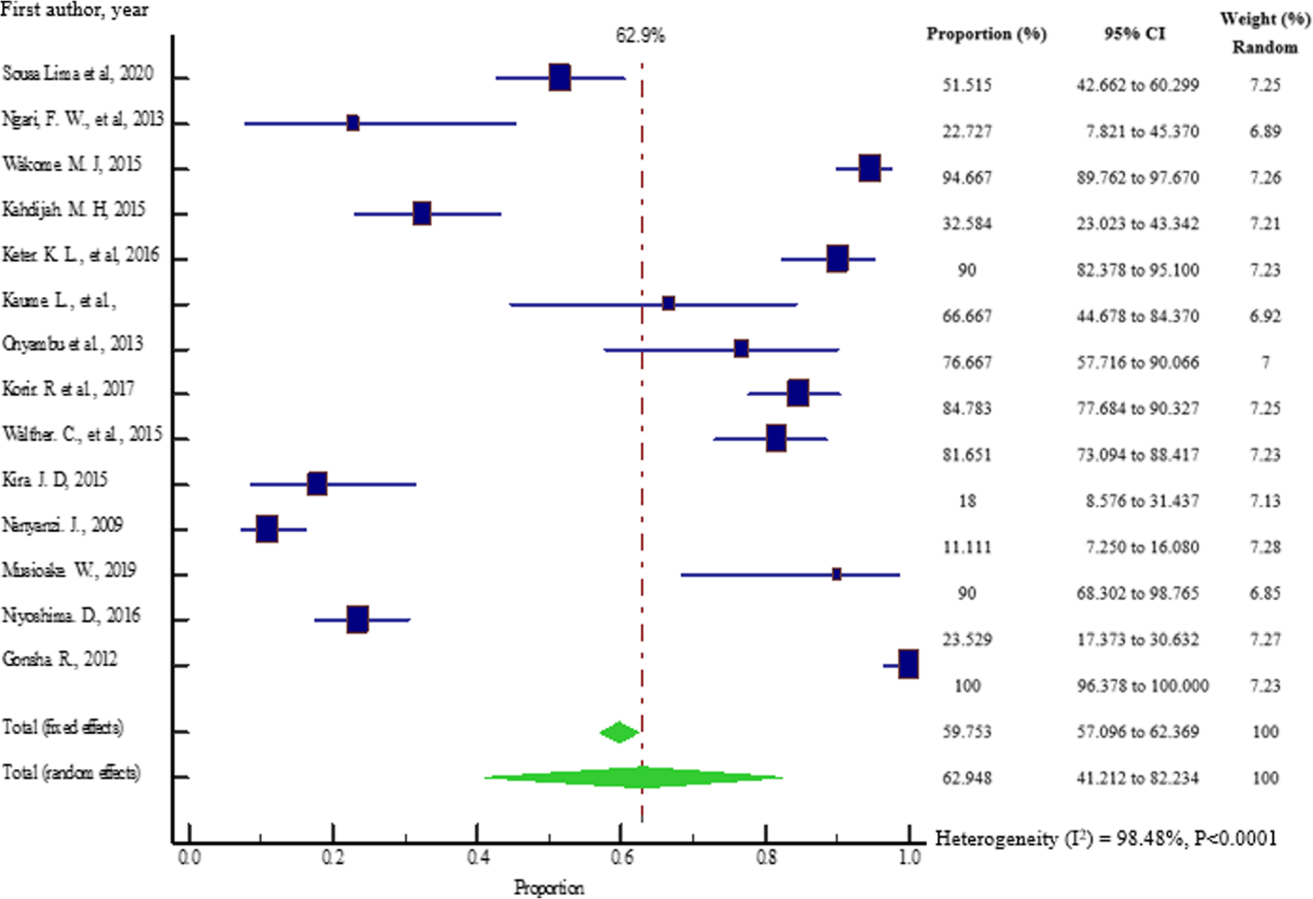
Pooled prevalence estimates of bacterial contamination in herbal medicines sold in East African countries from the year 2000 to 2020, using random effects model

In the East African countries, for the 14 eligible studies that were available, the prevalence of herbal medicines contaminated with bacteria as reported by individual studies varied widely. The prevalences ranged from 11.1% (95% CI = 7.250 to 16.080%) in a study conducted by Nanyanzi [20] in Uganda to 100% (95% CI = 96.378 to 100.000%) in a study done by Gonsha [30] in Uganda. The distribution of bacterial contaminants is shown in Fig. 5. The pooled prevalences of bacterial contaminants in herbal medicines are shown in Fig. 2.

**Fig. 3:**
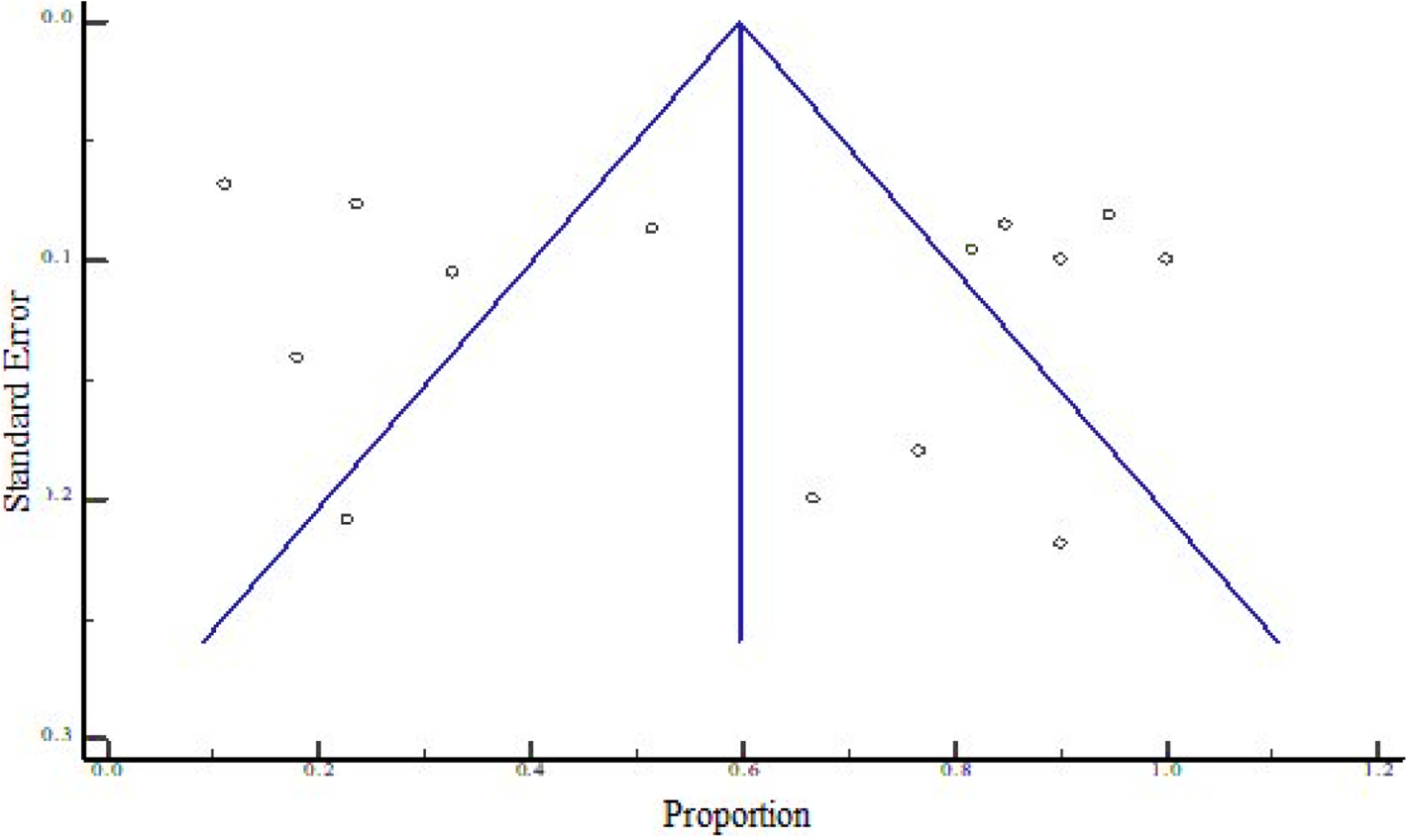
Bias assessment plot of studies that reported bacterial contamination in herbal medicines sold in East African countries from the year 2000 to 2020

The overall pooled prevalence of bacterial contaminants in the total sample of 1350 was 62.948% (95% CI = 41.212 to 82.234%), with heterogeneity (*I*^2^) of 98.48% (*P* < 0.0001) (Fig. 2). We constructed a funnel plot to analyze the publication bias. Despite the significant heterogeneity (*P* < 0.0001), the funnel plot displayed a symmetrical spread in terms of relative weight and effect size, hence demonstrating no evidence of publication bias in Fig. 3.

**Fig. 4:**
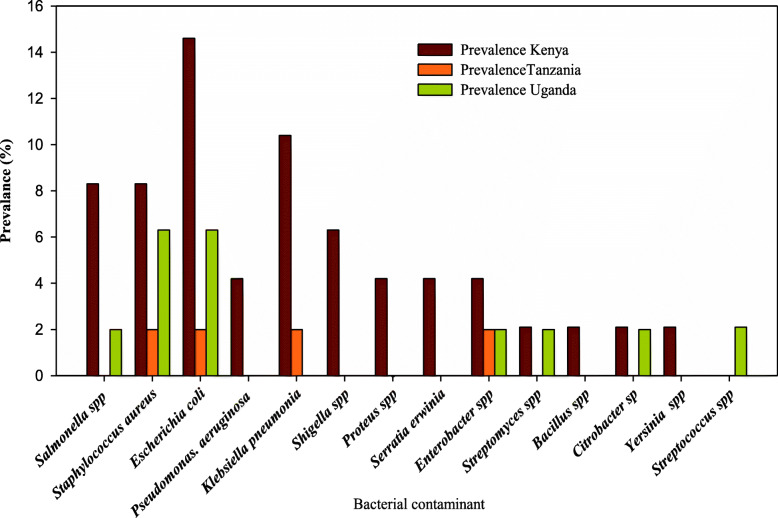
Distribution of potentially harmful bacterial contaminants in herbal medicines in East Africa from the year 2000 to 2020

A total of 48 potential clinically significant bacterial contaminants isolated from the HM belonged to fourteen genera. *Salmonella* spp. (5/48; 10.4%) was the most frequently reported primary pathogen, followed by *Shigella* spp. (3/48; 6.3%). Kenya reported the greatest number of bacterial contaminants (35/48; 72.9%), of which *E*. *coli* was the most reported (11/48; 23%) with the least reported being *Yersinia* spp., *Citrobacter* spp., *Bacillus* spp., and *Streptomyces* spp. (all at 2%; 1/48) (Fig. 4). Tanzania reported the least number of bacterial contaminants of herbal medicines (4/48; 8%), where *Klebsiella pneumoniae*, *Escherichia coli*, *Enterobacter spp*., and *Staphylococcus aureus* were the only reported genera (each at 2.1%; 1/48). Figure 4 shows the profiles of bacterial contaminants reported by the 14 research articles reviewed in this study.

### Meta-analysis of sub-groups

Since the eligible studies were highly heterogeneous, we subdivided the analysis into five sub-groups which included country of study, year of publication, routes of administration, diseases treated, and bacterial species isolated (Table 4). The microbes, *Bacillus spp*., *Yersinia spp*., and *Streptococcus spp*., which were reported in only one study, were excluded from the sub-group meta-analysis in the category of bacterial species isolated. In most of the categories adopted for sub-group analysis, heterogeneity (*I*^2^) remained high above the value (*I*^2^ = 98.48%, *p* < 0.0001) reported in the overall meta-analysis of all the studies (Fig. 2), except for Kenya (*I*^2^ = 96.51%, *p* < 0.0001) and Ulcer disease (*I*^2^ = 96.06%, *p* < 0.0001). As presented in Table 2, the trend was different in the sub-group category of “bacterial species isolated”, whereby heterogeneity (*I*^2^) reduced except for *Escherichia coli* (*I*^2^ = 98.53%, *p* < 0.0001).

**Table 4:** Sub-group analysis of pooled prevalence estimation of bacterial contaminants in herbal medicines sold in E. Africa from the year 2000 to 2020

Variable	Analysis				
	Number of studies	Prevalence % (95% CI)	***P*** value	***I***^**2**^ (%) (95% CI)	***P***_**het**_
**Country of study**					
Kenya	8	67.426(47.170 to 84.758) REF		96.51% (94.79 to 97.66)	< 0.0001
Tanzania	2	50.183(1.493 to 98.595)	0.0011	98.44% (96.56 to 99.29)	< 0.0001
Uganda	4	60.849(11.265 to 98.481)	0.11	99.3% (99.00 to 99.51)	< 0.0001
**Year of publication**					
2000 to 2015	9	59.190 (27.757 to 86.921)		98.76% (98.38 to 99.06)	< 0.0001
2016 to 2020	5	69.284 (39.342 to 92.254)	0.0041	98.06% (96.99 to 98.75)	< 0.0001
**Route of administration**					
Topical	2	76.619 (26.948 to 99.976) REF		98.75% (97.38 to 99.40)	< 0.0001
Not specified	5	57.453 (25.799 to 86.023)	0.0051	98.07% (97.01 to 98.75)	< 0.0001
Oral	9	65.947 (35.521 to 90.467)	0.0001	98.76% (98.37 to 99.05)	< 0.0001
**Disease treated**					
Not specified	9	72.849(49.577 to 90.891) REF		98.29% (97.70 to 98.73)	< 0.0001
HIV	2	48.294(17.905 to 79.405)	0.0004	88.87% (57.99 to 97.05)	0.0027
Cough	2	48.987(0.713 to 99.587)	0.0009	98.30% (96.19 to 99.25)	< 0.0001
Ulcers	2	62.077(9.259 to 99.575)	0.1114	96.06% (88.91 to 98.60)	< 0.0001
**Bacteria isolated**					
*Escherichia coli*	11	61.839(35.576 to 84.833) REF		98.53% (98.10 to 98.86)	< 0.0001
*Salmonella* spp.	5	37.653(16.424 to 61.75)	< 0.0001	96.13% (93.34 to 97.75)	< 0.0001
*Staphylococcus* spp.	9	68.572(44.285 to 88.399)	0.0239	97.87% (97.07 to 98.45)	< 0.0001
*Pseudomonas* spp.	2	38.66(14.352 to 66.491)	0.0002	84.83% (38.17 to 96.28)	0.0102
*Klebsiella* spp.	6	78.616 (60.095 to 92.484)	< 0.0001	96.11% (93.66 to 97.61)	< 0.0001
*Shigella* spp.	3	85.656 (80.908 to 89.603)	< 0.0001	43.8% (0.00 to 83.19)	0.1687
*Proteus* spp.	2	86.774(81.822 to 90.789)	< 0.0001	25.96% (25.96 to 25.96)	0.2452
*Serratia* spp.	2	86.774(81.822 to 90.789)	< 0.0001	25.96% (25.96 to 25.96)	0.2452
*Enterobacter* spp.	4	87.575(70.410 to 97.903)	< 0.0001	93.71% (87.09 to 96.94)	< 0.0001
*Citrobacter* spp.	2	94.893 (70.404 to 98.584)	< 0.0001	96.60% (90.80 to 98.74)	< 0.0001

All the *p* values of sub-group prevalence with respect to the group reference were < 0.05, except for Uganda and Ulcers

**Fig. 5:**
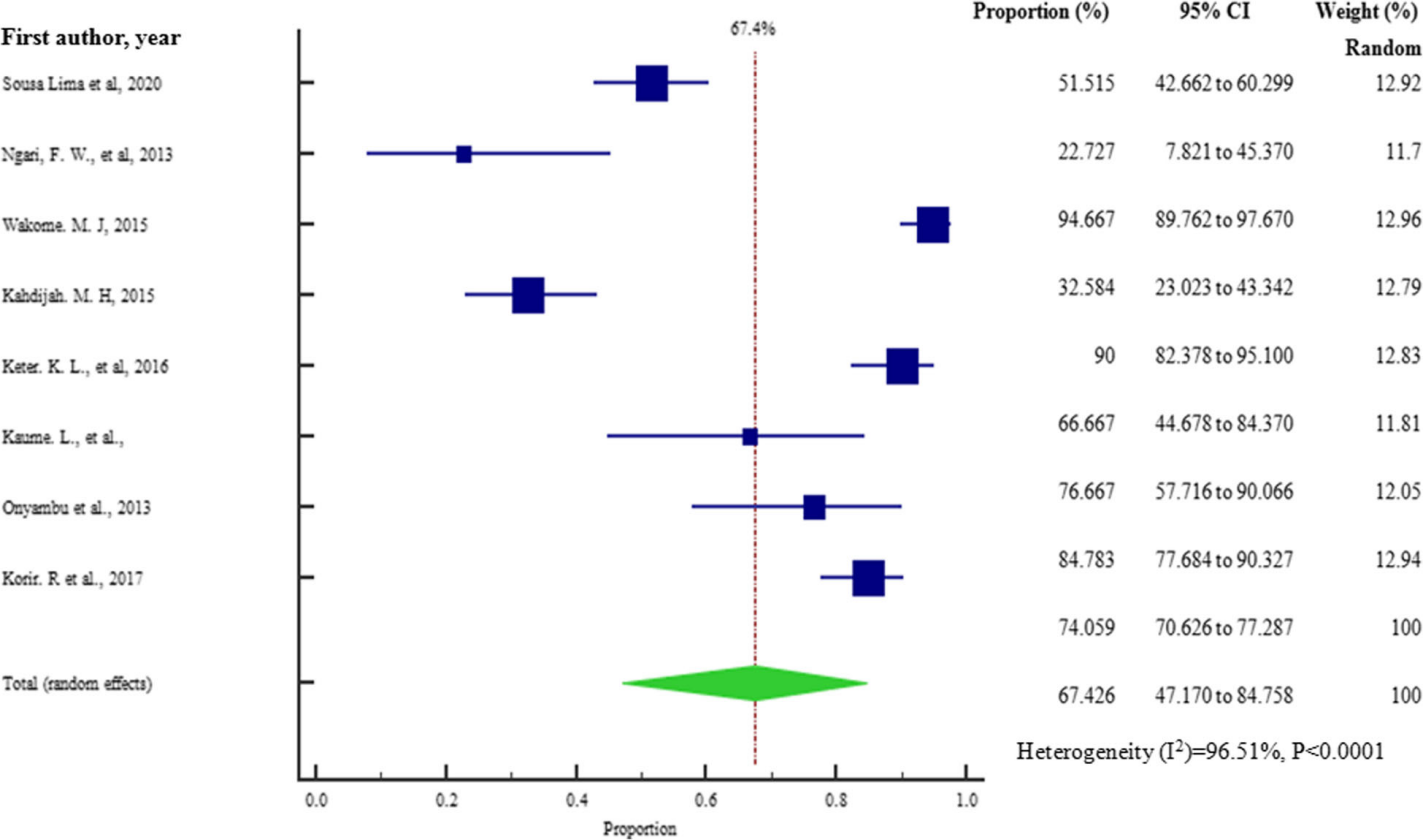
Pooled prevalence estimates of bacterial contamination in herbal medicines sold in Kenya from the year 2000 to 2020, using random effects model

**Fig. 6:**
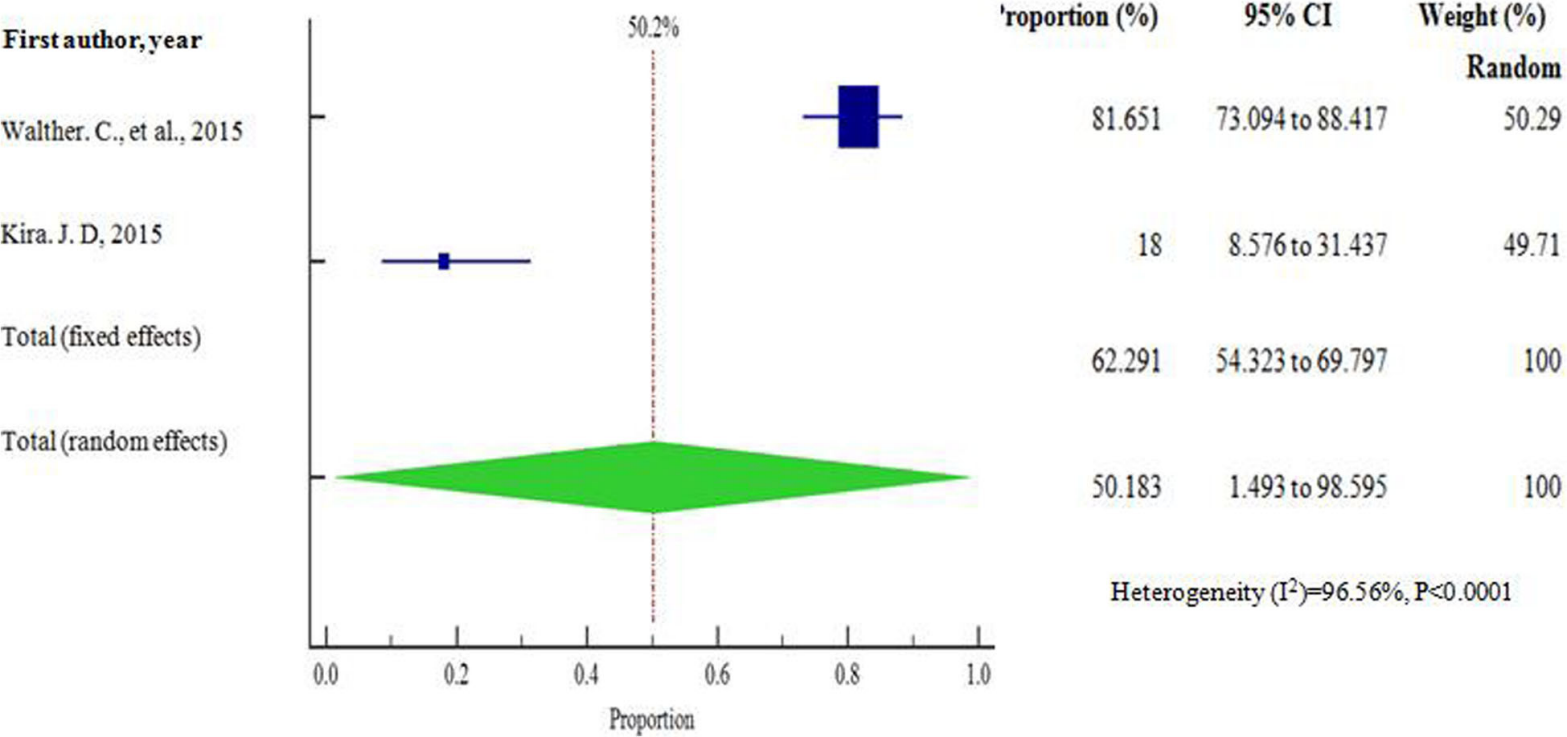
Pooled prevalence estimates of bacterial contamination in herbal medicines sold in Tanzania from the year 2000 to 2020, using random effects model

**Fig. 7:**
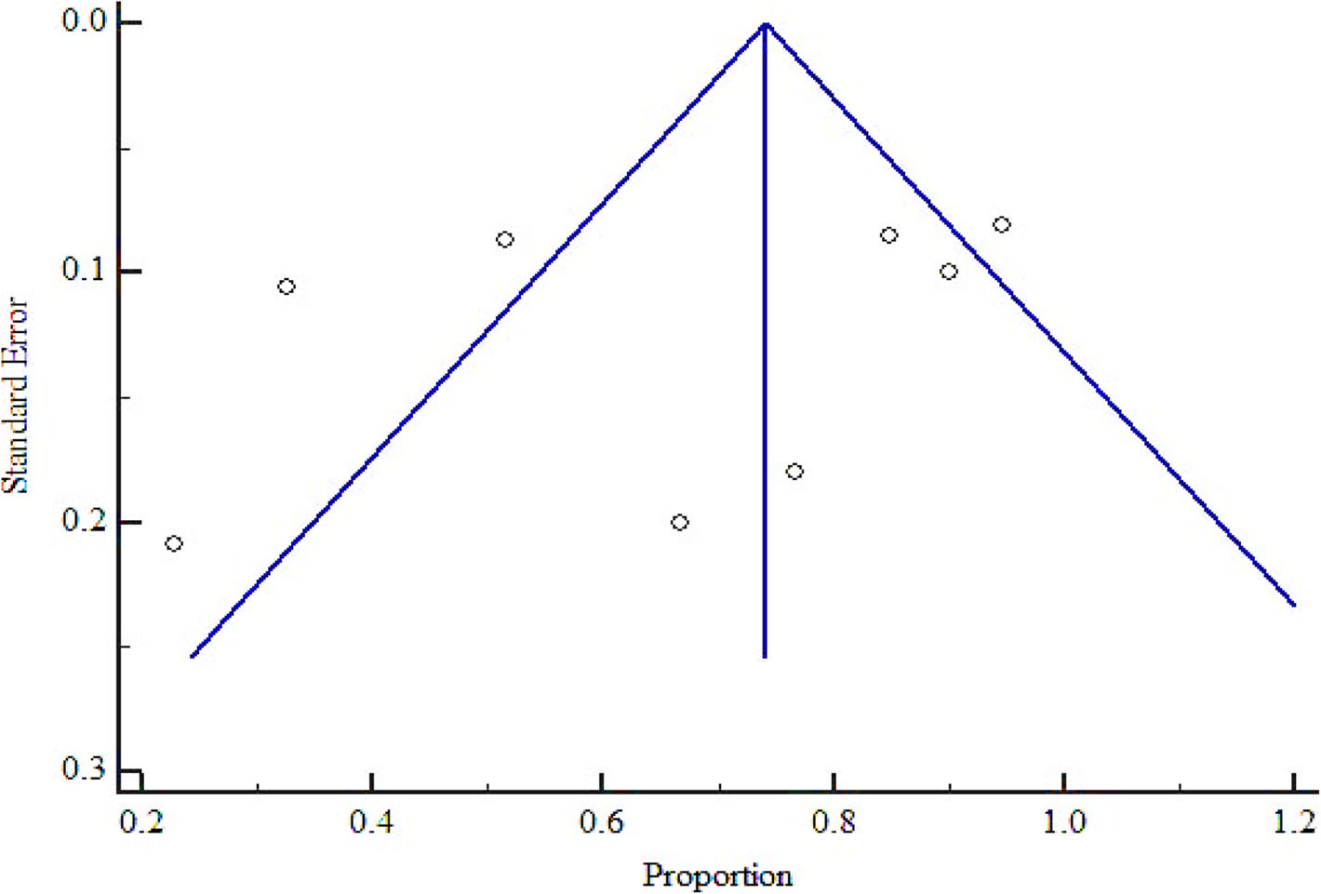
Bias assessment plot of studies that reported bacterial contamination in herbal medicines sold in Kenya from the year 2000 to 2020

**Fig. 8:**
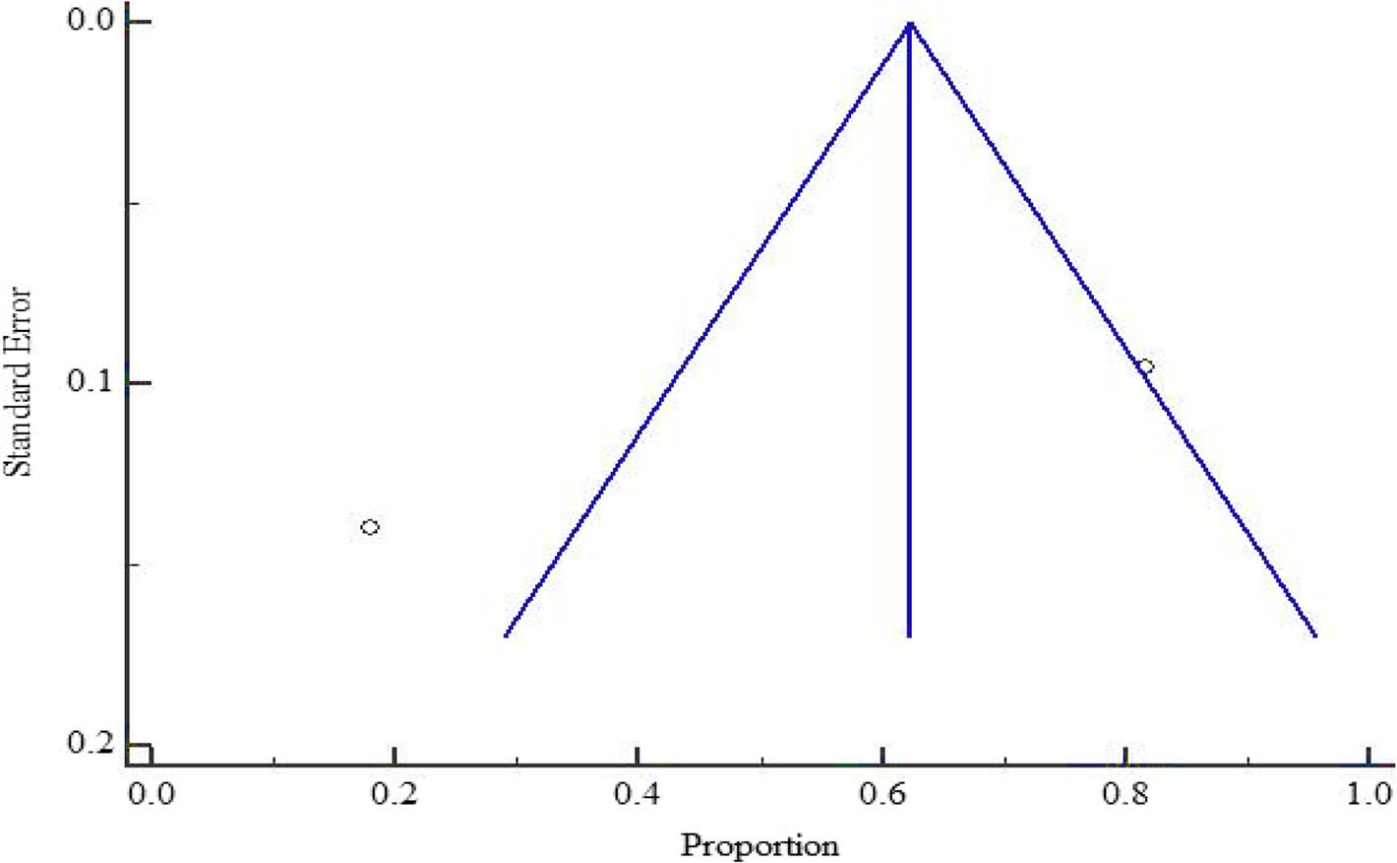
Bias assessment plot of studies that reported bacterial contamination in herbal medicines sold in Tanzania from the year 2000 to 2020

**Fig. 9:**
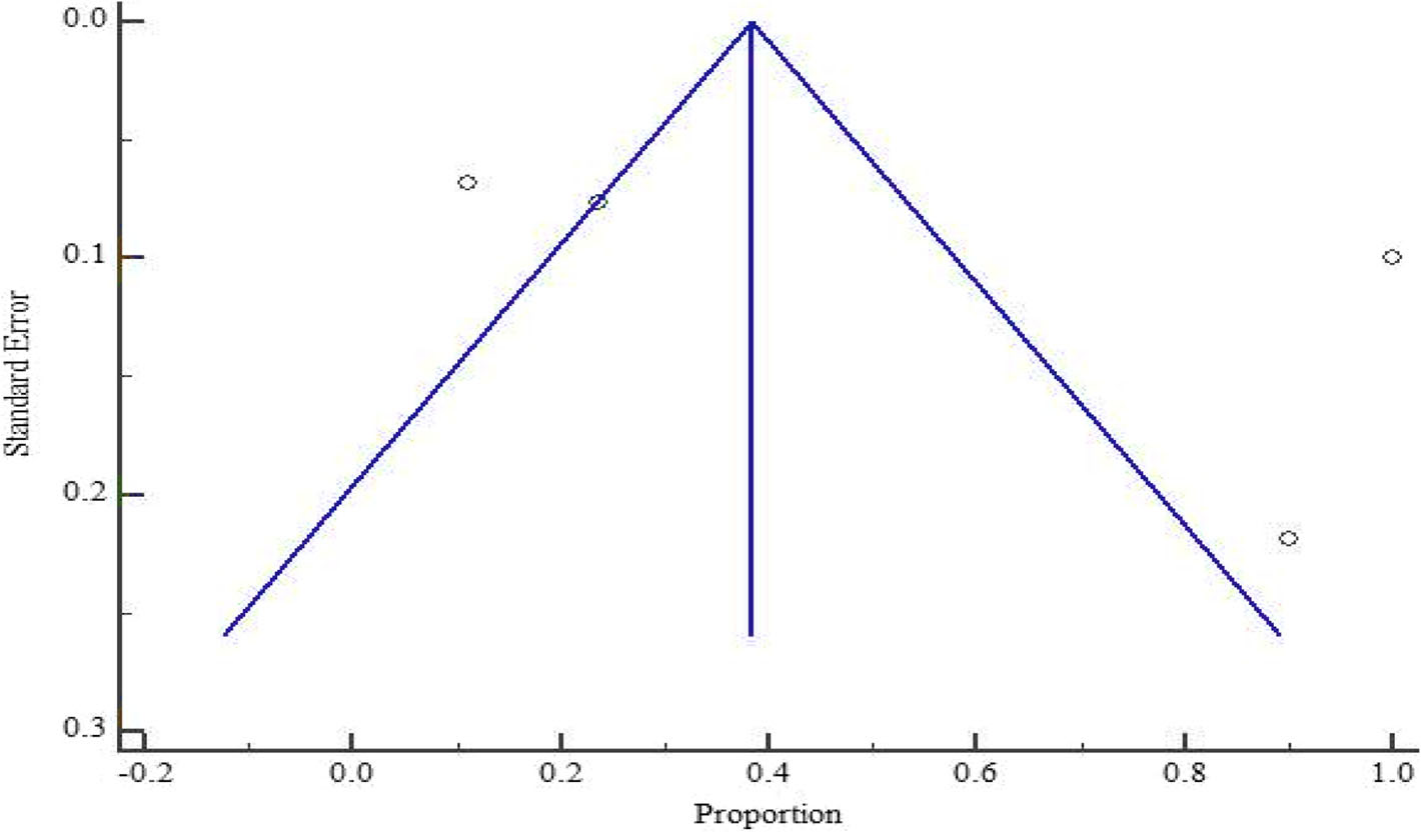
Bias assessment plot of studies that reported bacterial contamination in herbal medicines sold in Uganda from the year 2000 to 2020

**Fig. 10:**
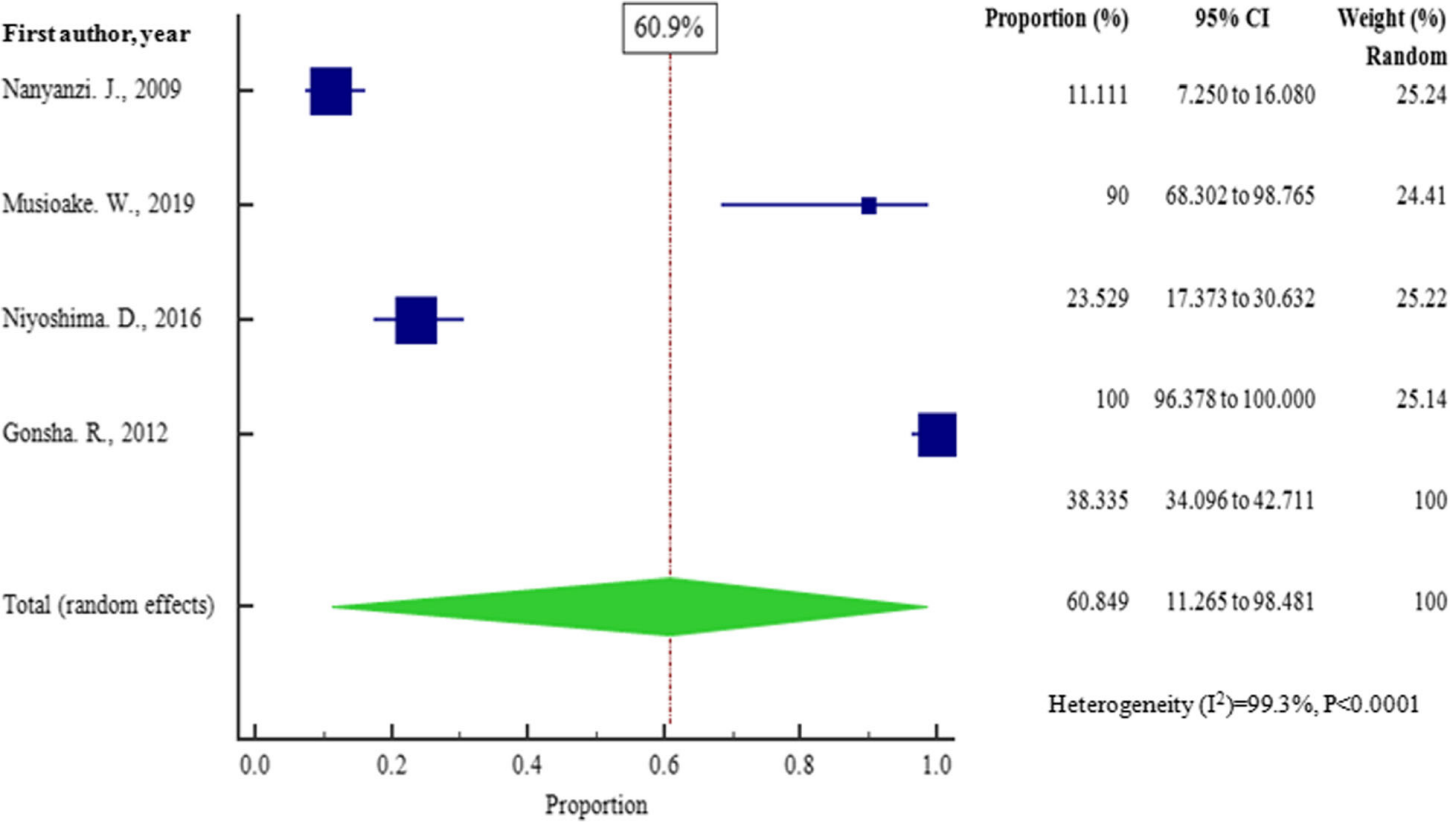
Pooled prevalence estimates of bacterial contamination in herbal medicines sold in Uganda from the year 2000 to 2020, using random effects model

As presented in Figs. 5 and 6, at country level, the highest and lowest pooled prevalences of bacterial contaminants in commercial herbal medicine were reported in Kenya, 67.426% (95% CI = 47.170 to 84.758%) and Tanzania 50.183% (95% CI = 1.493 to 98.595%), respectively. There was no evidence of publication bias in the three countries (Uganda, Kenya and Tanzania), as exhibited by the symetrical nature of the graphs (Figs. 7, 8 and 9). In Uganda, the pooled prevalence was 60.9% (95% CI = 11.256 to 98.481%) as shown in Fig. 10. Table 4 shows that the pooled prevalence in Kenya was significantly different from that in Uganda (*p* = 0.0011) but not from that in Tanzania (*p* = 0.11).

Pertaining to the variation of bacterial prevalence rates by years of publication, the period between the year 2016 to 2020 (*n* = 5) registered the highest pooled prevalence of bacterial contaminants, 69.284% (95% CI = 39.342 to 92.254%) as compared to 59.190% (95% CI = 27.757 to 86.921%) reported during the period 2000 to 2015 (*n* = 9). The prevalences were significantly different (*p* = 0.0041), during the two periods (Table 2). Regarding the analysis by route of administration, the herbal medicines administered topically (*n* = 2) contained the greatest pooled prevalence of bacterial contaminants 76.619% (95% CI = 26.948 to 99.976%) while the lowest prevalence, 57.453% (95% CI = 25.799 to 86.023%) was reported among the herbal medicines in which the published studies did not specify the route of administration (*n* = 5). The bacterial prevalence was significantly different between topical and oral therapies (*p* = 0.0001) as well as the therapies with unspecified administration routes (*p* = 0.0051). The most frequently isolated bacteria from oral herbal medicines were *Escherichia coli*, *Shigella* spp., *Serratia* spp., *Proteus* spp., *Salmonella* spp., and *Staphylococcus aureus*, while from topical herbal medicines, they were *Klebsiella pneumonia*, *Pseudomonas aeruginosa*, and *Escherichia coli*. Most of these bacteria are implicated in causing respiratory as well as gastrointestinal complications in man [31]. We also analyzed the prevalence of bacterial contaminants in herbal medicines with regard to the types of diseases they treat. The highest bacterial prevalence, 72.849% (95% CI = 49.577 to 90.891%) was observed in therapies for which the diseases treated were not stated in the respective publications (*n* = 9), while the lowest pooled bacterial prevalence, 48.294% (95% CI = 17.905 to 79.405%) was reported in herbal medicines vended for management of HIV complications (*n* = 2). Based on species of the bacterial contaminants, *Citrobacter* spp. had the highest pooled prevalence, 94.893% (95% CI = 70.4 to 98.6%), followed by *Enterobacter* spp., 87.575% (95% CI = 70.4 to 97.9%), as well as *Proteus* spp., and *Serratia* spp., both at 86.8% (95% CI = 81.8 to 90.8%). *Pseudomonas* spp. had the least pooled prevalence of 38.66% (95% CI = 14.4 to 66.5%). *Escherichia coli* was reported by the greatest number of studies (*n* = 11) and had a pooled prevalence of 61.839% (95% CI = 35.6 to 84.8%); this was significantly different from the pooled prevalence of all the other bacteria (Table 4).

### Meta-regression

**Fig. 11:**
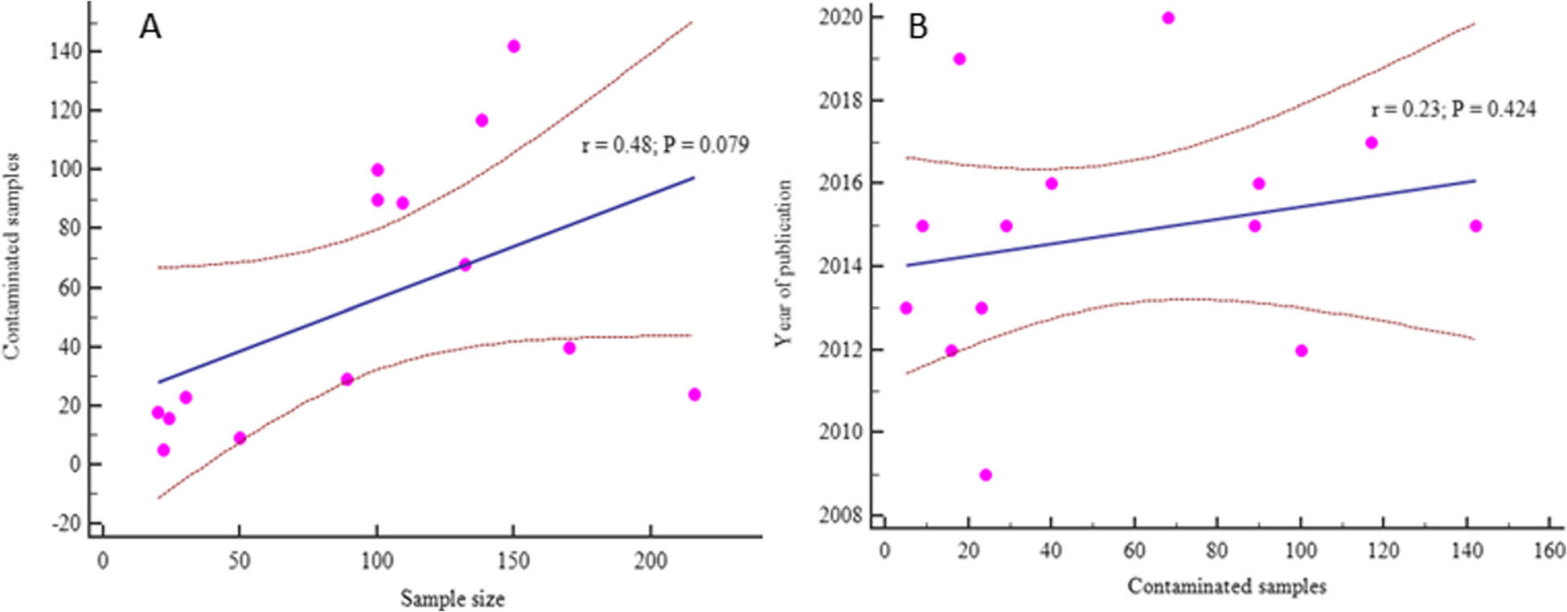
Meta-regression analysis by sample size and contaminated samples (**a**) and by year of publication and bacteriologically contaminated HM samples (**b**), sold in E. Africa from 2000 to 2020

Meta-regression analysis was performed to examine the continuous variables of contaminated HM samples and sample size (*p* = 0.079) as well as year of publication (*p* = 0.424). The results showed that the sample sizes and years of publication were not significantly associated with pooled prevalence of contaminated samples (*p* > 0.05) (Fig. 11).

### Sensitivity analysis

**Fig. 12:**
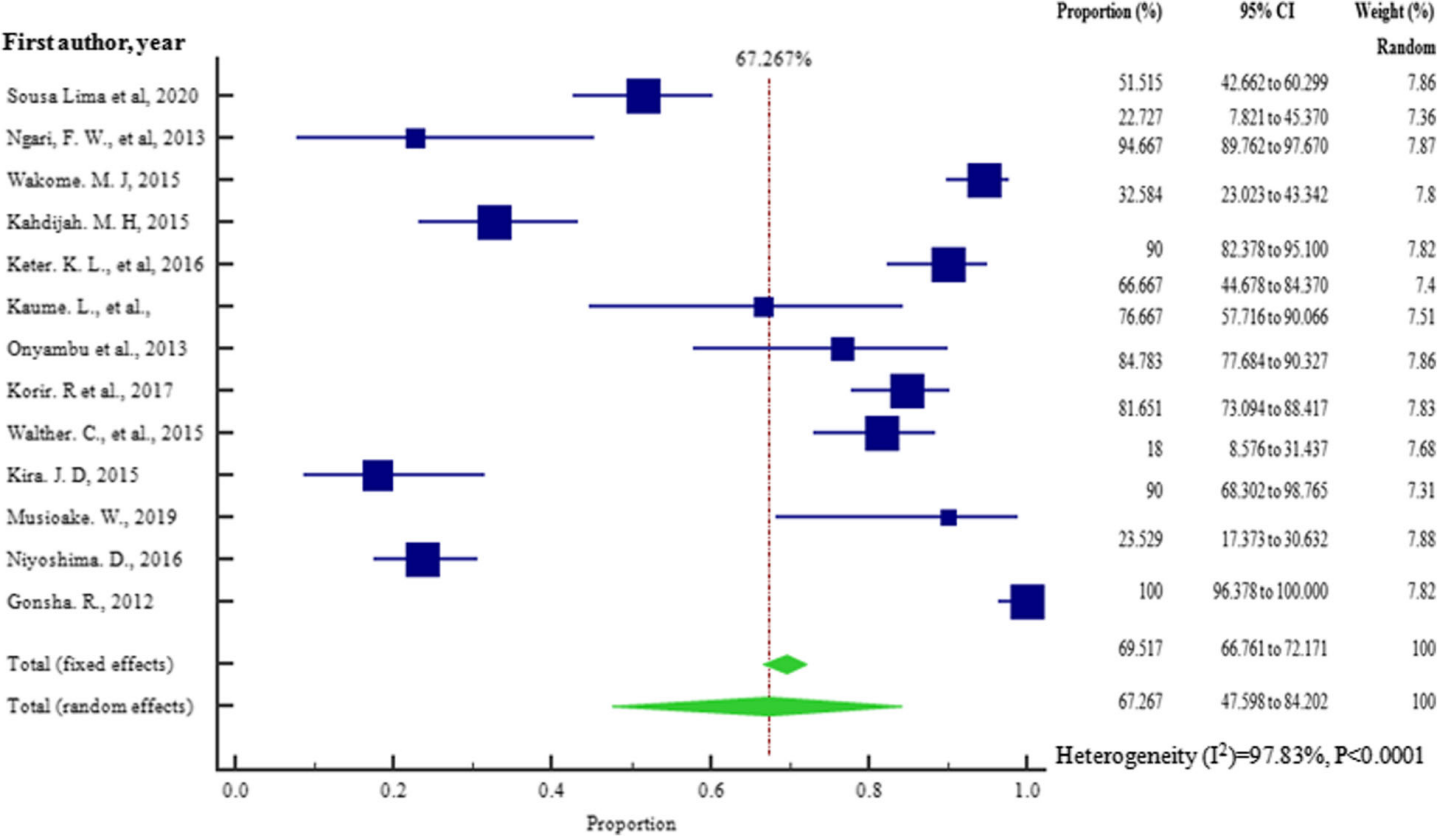
Forest plot showing sensitivity analysis of the pooled prevalence of bacterial contamination in herbal medicines sold in Tanzania from the year 2000 to 2020

Sensitivity analysis involved removal of one study which had the largest sample size [15]. Figures 2 and 12 show that there was a slight increase in the overall pooled prevalence of bacterial contaminants, from the original 62.948% (95% CI = 41.212 to 82.234%) to 67.267% (95% CI = 57.598 to 84.202%) respectively, with heterogeneity *I*^2^ = 97.83% (95% CI = 97.17 to 98.33%), *p* < 0.0001.

## Discussion

We report a total of fourteen original scientific studies that investigated the prevalence of bacterial contaminants in commercial herbal medicines in East Africa and published the findings online from the year 2000 to 2020. The fourteen studies were conducted in Kenya, Uganda, and Tanzania. No eligible studies were found in Rwanda, Burundi, and Southern Sudan and therefore the need for more adequate research on this subject. Findings from those studies will help to circumvent the widespread safety concerns linked to the extensive consumption and trade of HM in the East African region and beyond [8, 20, 32]. The total sample size from 14 studies was 1350 of which 770 (57%) samples were positive for bacterial contamination. The studies were highly heterogeneous (*I*^2^ > 84%, *P* < 0.05) but heterogeneity was reduced for bacterial isolates including Shigella, Proteus, and Serratia (*I*^2^ < 45%, *P* > 0.05), and there was no demonstrable evidence publication bias. The overall pooled prevalence of all the bacterial species was 62.948%. Although *Escherichia coli* represented the most prevalent contaminant (23%), *Salmonella* spp. was the most frequently reported primary pathogen with a pooled prevalence of 10.4%, followed by *Shigella* spp. (6.3%). The high prevalence of *E*. *coli* is indicative of the grossly deprived environmental hygiene associated with fecal contamination of water, plants, and other resources that humans consume in the E. African region. The abundant presence in HM, of primary pathogens such as *Salmonella* spp. and *Shigella* spp. is of great clinical significance because these have been implicated in deadly outbreaks of diarrheal diseases such as typhoid fever in the East African region [33, 34]. According to the World Health Organization, such microbes should have zero (0%) presence in HM [35]. The major limitation in studies selected for this meta-analysis was the fact that phenotypic methods were used in characterization of the bacterial contaminants instead of molecular approaches. This may have hampered effective identification of the isolates, hence affecting the prevalences reported. Our meta-analysis revealed that the most frequently isolated bacterial contaminants of commercial HM in East Africa are somewhat different from those commonly isolated elsewhere. For example, in Southern Africa, *Bacillus* spp., *Pantoea* spp., *Rahnella aquatilis*, and *Acinetobacter baumannii* represented some of the commonest bacterial HM contaminants [16]. However, the common herbal contaminants such as *Salmonella* spp. and *Escherichia coli* reported in recent systematic reviews in parts of Europe and Asia were in tandem with those observed in our meta-analysis in the E. African region [36]. Though not commonly observed among the HM contaminants in East Africa, most bacterial contaminants such as *Rahnella aquatilis* that were reported in Southern Africa are of potential risk to public health since they are abundant in soil, water, and most livestock, yet these bacteria are associated with human diseases [20, 21]. Differences in prevalence and dynamics of bacterial contaminants of HM in different regions around the world may be partly explained by variations in climate and/or geographical seasons at the time of sampling, formulation, and implementation of herbal safety policies, as well as the hygiene status in the different regions.

### Limitations of the study

This study was limited by the low number of research articles published and available online on the topic of bacterial contamination of herbal medicines sold in the East African member countries (EAC), the small number of countries (only 3 of the 6 countries of EAC) that contained the eligible studies, and the language (only English studies were available online among the eligible studies).

### Conclusions and recommendations

The pooled prevalence of potentially harmful bacterial contaminants in herbal medicines sold in East African countries from the year 2000 to 2020 was 62.948%. *Escherichia coli* was the most prevalent contaminant, indicating a possible fecal contamination among HM samples sold in this region. *Salmonella* spp. and *Shigella* spp. were the most frequently reported primary pathogens with a pooled prevalence of 10.4 and 6.3%, respectively. This implies that commercial HM pose considerable health risks to communities across E. Africa by disseminating clinically significant microbes. The selected studies were conducted in Kenya (57%, 8/14), Uganda (29%, 4/14), and Tanzania (14%, 2/14). The burden remains unclear in the rest of the East African countries (Rwanda, Burundi, and South Sudan), where eligible studies were not available. Adequate research pertaining to microbial safety of herbal medicines in East African Community member countries remains highly desired. We recommend establishment of strong and concerted herbal safety mechanisms in Eastern Africa, to improve public health protection in the region.

## Data Availability

Datasets generated and analyzed during this meta-analysis are available from the corresponding author on request.
